# Supplementary material for: Computationally designed stem-epitope mimetics elicit broadly reactive antibodies
Source: bioRxiv. 2025 Jan 22:2025.01.22.634229. Preprint. [Version 1] doi: 10.1101/2025.01.22.634229 (PMC12191098; doi:10.1101/2025.01.22.634229)
Supplement: Supplement 1 [file media-1.pdf]

Supplementary Materials

Computationally designed stem-epitope mimetics elicit broadly reactive antibodies

Sarah Wehrle[#], Andreas Scheck[#], Laura Reusch[#], Flavio Matassoli, Sandrine Georgeon, Karla M. Castro, Johannes Cramer, Wayne Harshbarger[5] Stéphane Rosset, Sarah F. Andrews, Karin Schön, Badiaa Bouzya, Ronan Rouxel, Normand Blais, Enrico Malito, Adrian McDermott, Thomas Krey, Corey P. Mallett, Ventzislav Vassilev, Davide Angeletti[*], Bruno E. Correia[*]

[#]These authors contributed equally

*Correspondence: davide.angeletti@gu.se (D.A.), bruno.correia@epfl.ch (B.E.C.).

Supplementary Figures

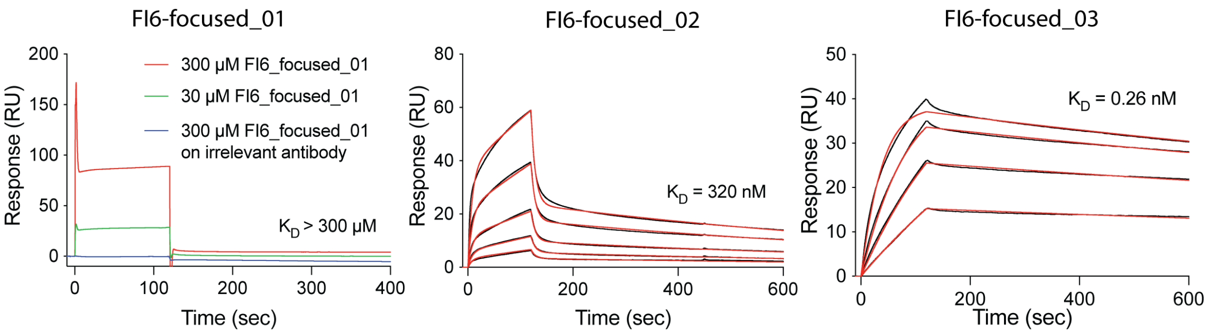

Figure S1. ***Affinities of FI6-focused intermediate designs to FI6 antibody measured by SPR.*** *Affinity of the initial FI6-focused_01 design was too low to determine a* K_D. *The irrelevant antibody used as negative control was an RSV specific antibody (D25). For the SPR measurements of FI6-focused_01 design FI6 IgG was immobilized on the chip and design was flown.For the SPR measurements of FI6-focused_02 and _03 design, designs were immobilized and FI6 Fab was added.*

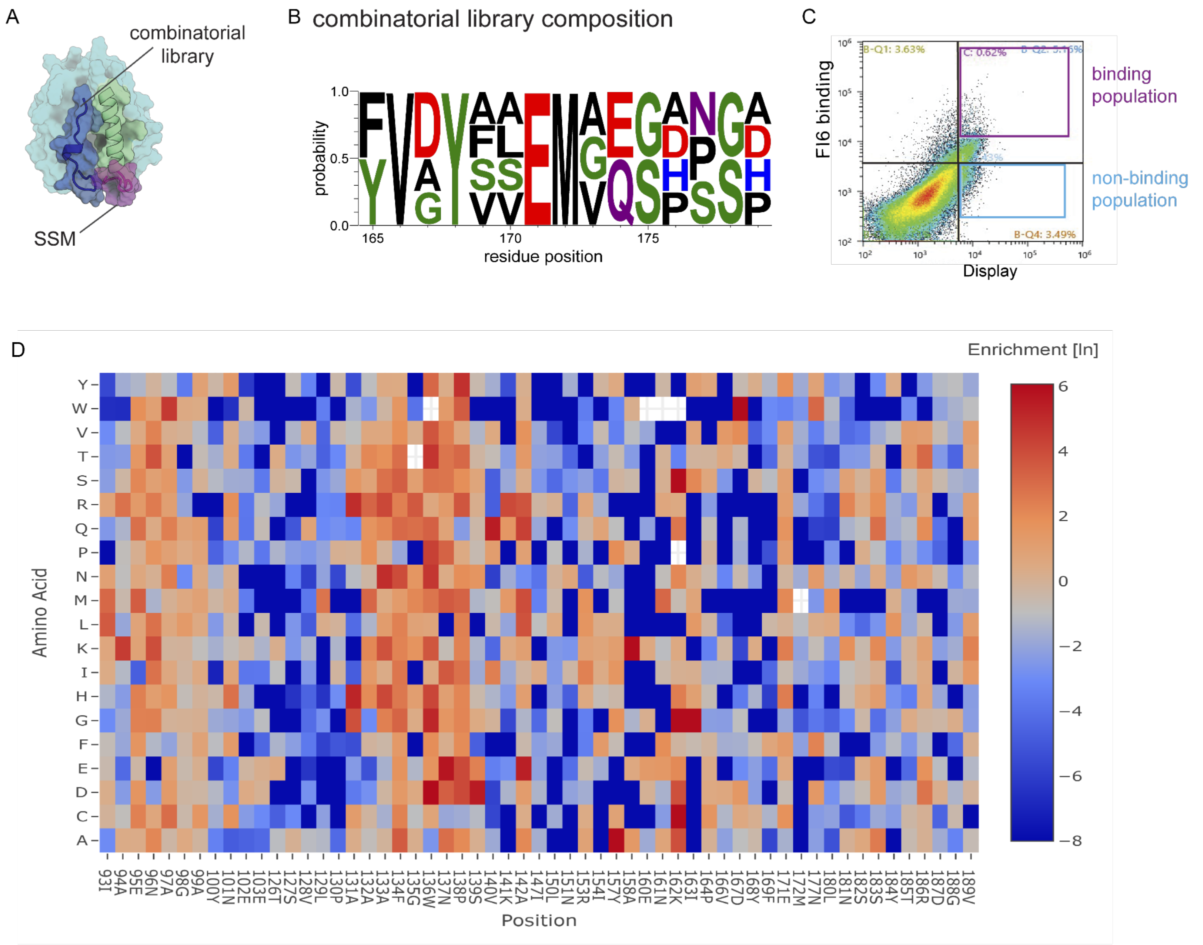

$$\text{Enrichment} = \ln\left(\frac{\text{frequency in binding population}}{\text{frequency in non binding population}}\right)$$

*Figure S2. **Overview of libraries for FI6-focused design affinity maturation. A.** FI6-focused design with targeted structural elements highlighted (green = grafted epitope helix, blue = hydrophobic pocket, purple = loop connecting the epitope helix to scaffold). The hydrophobic pocket was targeted with the combinatorial library. The connecting loop was targeted with the SSM library. **B.** Logo plot of positions targeted in combinatorial library. The library was sorted three times with decreasing concentrations of the FI6 antibody. **C.** Density plot of SSM library. Constructs binding strongly to the FI6 binding antibody and displaying on the yeast surface were sorted as binding population. Constructs without FI6 binding but displaying on yeast were sorted as non-binding population. **D.** Heat map of residues enriched in the binding population over the non-binding population.*

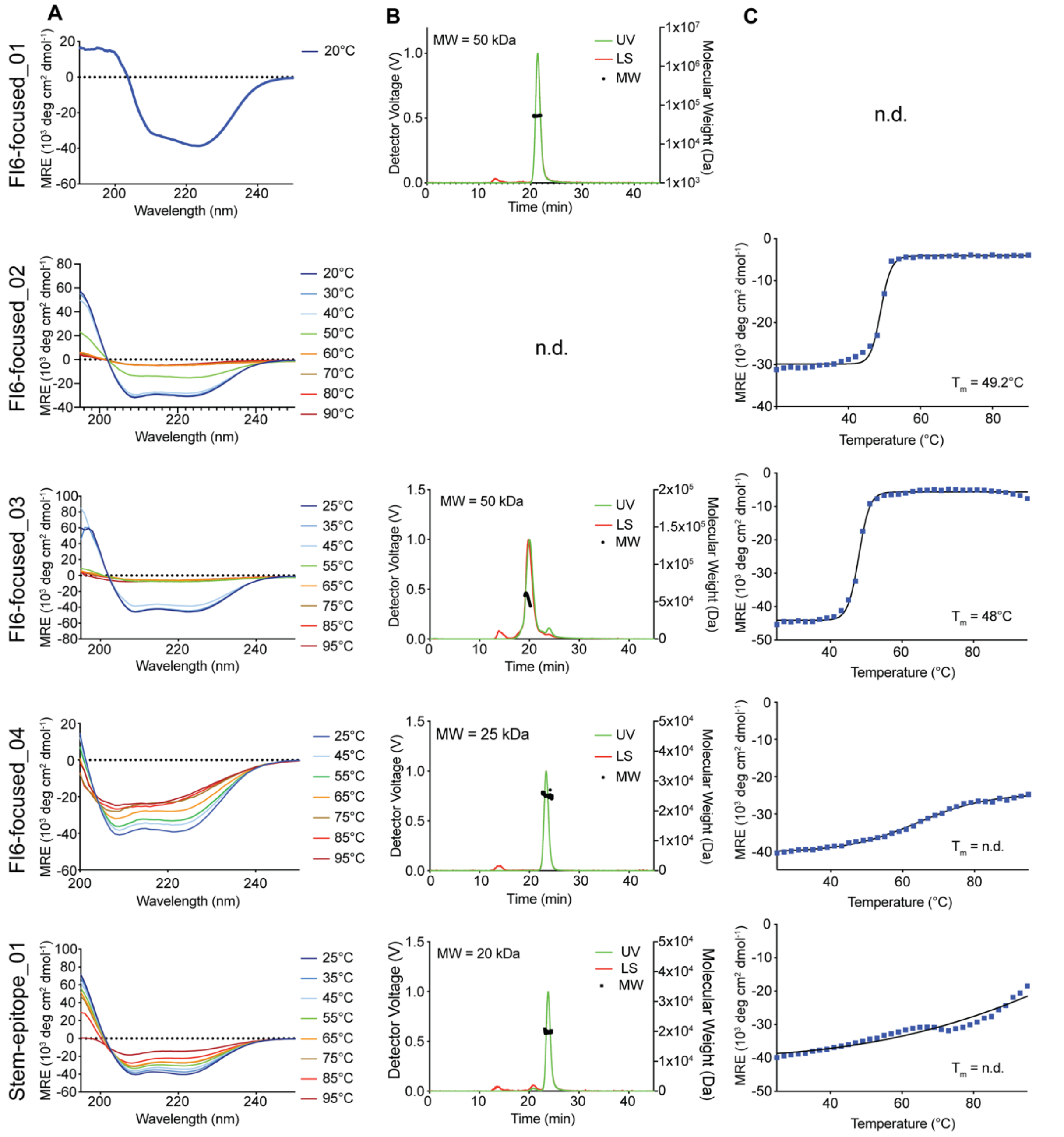

Figure S3. ***Biophysical characterization of relevant designs.*** *A. Circular dichroism (CD) spectra at indicated temperatures. B. Size exclusion chromatography coupled to multi-angle light scattering. C. Thermal denaturation curves. Designed proteins were melted from 20 to 90 °C by CD. The melting temperature (T_m) was determined by the change of ellipticity at the global curve minimum (208 nm).*

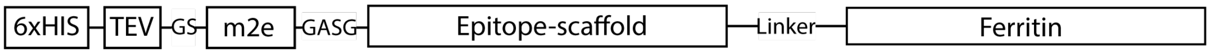

Figure S4. **Schematic overview of nanoparticle construct.** Epitope-scaffolds were fused C-terminally to ferritin separated by a GS-linker. They were labelled N-terminally with a His-tag for purification and a TEV cleavage site. The m2e T cell epitope was introduced between epitope-scaffold and His-tag.

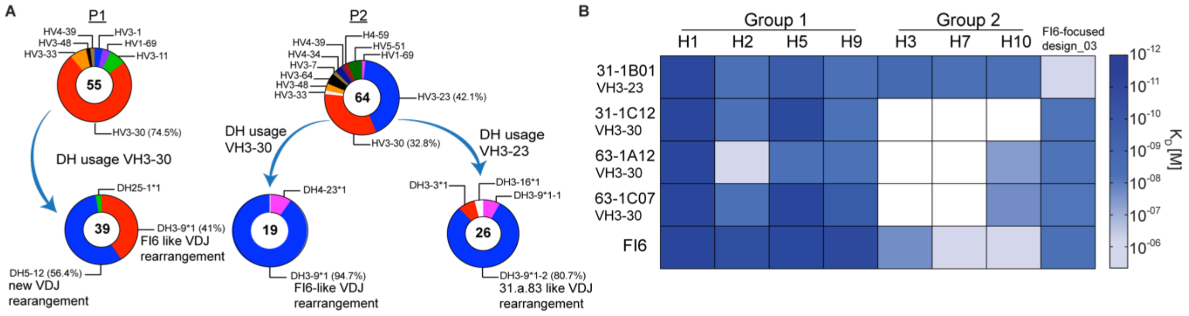

Figure S5. ***Analysis of human antibodies cross-reactive to FI6-focused design and H1. A.*** *Analysis of VDJ rearrangement pulled down antibodies and comparison to known bnAbs. B. Binding breadth and affinity of the different pulled down antibodies compared to FI6.*

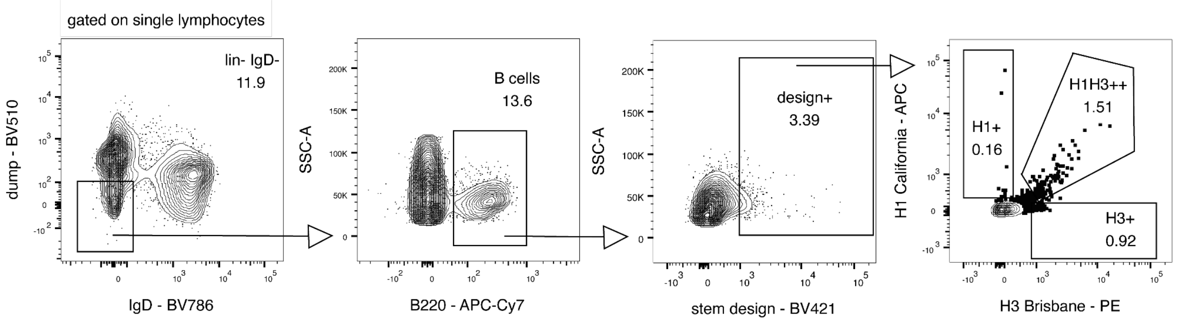

Figure S6: ***Gating strategy for B cell staining after immunization with 3x stem design. A.*** *Gating strategy to identify stem-design binding and H1-H3 cross-reactive B cells after immunization with 3x stem design. H1+H3+ cross-reactive B cells are selected as lin- IgD- B220+ design+.*

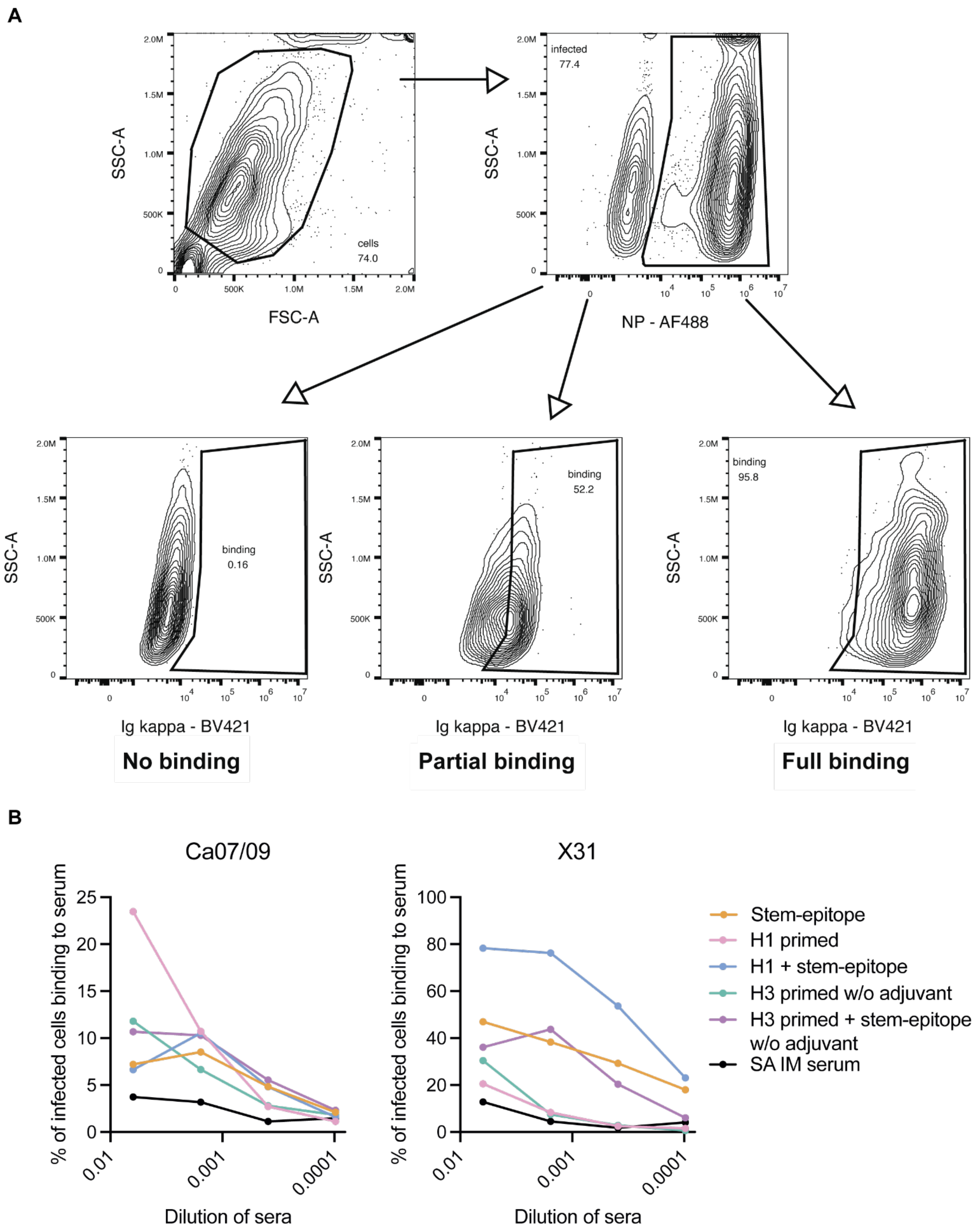

*Figure S7. **A.** Gating strategy to infected cells binding to serum. Gating strategy to identify serum binding to infected cells using flow cytometry. Analyzed are NP+ Ig kppa+ cells. **B.** Percentage of infected cells binding to serum (antibody).* Frequency of infected cells binding to sera from immunized mice. Shown are 4-fold dilutions.

*Table S1. **Amino acid sequences of relevant designs and segments of the nanoparticle constructs.***

Name	Sequence
m2e	SLLTEVETPIRNEWGSRSNDS
Linker	GGSGGSGGSGGSNGTGGSGGS
Ferritin	SKDIEKLLNEQVNKEMQSSNLYMSMSSWCYTHSLDGAGLFLFDHAAEEYEHAKK LIIFLNENNVPVQLTSISAPEHKFEGLTQIFQKAYEHEQHISESINNIVDHAIKSKDHA TFNFLQWYVAEQHEEEVLFKDILDKIELIGNENHGLYLADQYVKGIAKSRKS
FI6-focused design_01	SDAQKQDWGNLKRYAEANKELVRKGKQKDRVVFMGNSITEGWVANDAAFFED NGYVGRGIGGQTSSHFLLRFREDVIKLAPALVVINAGTNDIAENAGAYNEEYTFGN IVSMVELARANKIKVILTSVLPAAAFGWNPSVKKATQAIIDLNTRIRNYAIENKIPYV DYFAEMVEGDNAALNSSYTRDGVHPTLEGYKVMEALIKKAIDKVL
FI6-focused design_02	SDAQKQDWGNLKRYAEANKELVRKGKQKDRVVFMGNSITEGWVANDAAFFED NGYVGRGIGGQTSSHFLLRFREDVIKLAPALVVINAGTNDIAENAGAYNEEYTFGN IVSMVELARANKIKVILTSVLPAAAFGWNPSVKAATQAIINLNTRIINYAIENKIPFV DYFVEMAQSPNGDLNSSYTRDGVHPTLEGYKVMEALIKKAIDKVL
FI6-focused design_03	SDAQKQDWGNLKRYAEANKELVRKGKQKDRVVFMGNSITEGWVANDAAFFED NGYVGRGIGGQTSSHFLLRFREDVIKLAPALVVINAGTNDIAENAGAYNEEYTFGN IVSMVELARANKIKVILTSVLPHAAAGWNESQKEATQAIINLNTRIINYAIENKIPFV DYFVEMAQSPNGDLNSSYTRDGVHPTLEGYKVMEALIKKAIDKVL
FI6-focused design_04	SDAEKQDPANKKRYAEANKELVRKGKQKNRVVFMGNSITEGWVANDPAFFED NGYVGRGISGQTSSHMLERFEEDVIKLKPAVVVIMAGTNDIAENAGPYNEEQTFG NIVKMVELARAAKIKVILTSVLPHAAAPWNESQKEATQAIINLNTRIINYAIENKIPFV DYFVEMAQSPNGDLNSSYTRDGVHPNLEGYKVMEALIKKAIDKVL
Stem-epitope design_01	GEPEVTDQLEWQSNQPWEQALNRFWDYLRWVQTLSDQVQEELQSSQVTQEL TALMEDTLTEAIAYMKELEEQLGPVAEETRLKLTQNVIDAITNLVNDMAELRNRL GQYRNEVHTMLGQSTEEIRARLSTHLRKMRKRLMRDAEDVQKALAVYKAGA

*Table S2. **X-ray data collection and refinement statistics***

	**Stem-epitope in complex with FI6 Fab**	**FI6-focused_03**	**FI6_focused_03 FI6**	**FI6-focused_04 FI6**
**PDB ID**	8UZP	9HI5	9HI4	9HI6

**Data Collection**				
Wavelength (λ)	1	0.9762	0.9801	1.037
Resolution range (Å)	37.79 – 2.71 (2.80 – 2.71)	43.48 - 1.9 (1.968 - 1.9)	44.84 - 1.7 (1.761 - 1.7)	39.22 - 1.501 (1.555 - 1.501)
Space group	C2	P 1	P 1 21 1	P 1 21 1
Cell dimensions				
a, b, c (Å)	269.0, 55.9, 92.0	49.05, 63.31, 98.18	91.23, 78.14, 118.72	71.213, 47.869, 107.093
α, β, γ (°)	90, 90, 90	96.356, 99.528, 114.57	90, 100.601, 90	90, 106.126, 90
Total reflections	125034 (12471)	280565 (28234)	1250953 (127116)	759197 (74564)
Unique reflections	36153 (2434)	79483 (7867)	180128 (17937)	110620 (10830)
Multiplicity	3.5 (3.7)	3.5 (3.6)	6.9 (7.1)	6.9 (6.9)
Completeness (%)	90.7 (65.1)	97.09 (95.43)	99.88 (99.88)	99.58 (98.19)
I/σI	13.4 (2.9)	12.38 (1.89)	14.27 (1.08)	11.78 (1.35)
Wilson B-factor	44.60	30.57	27.18	19.50

R-merge	0.142 (0.678)	0.05935 (0.5936)	0.07322 (1.642)	0.09495 (1.155)
$CC_{1/2}$	0.81 (0.65)	0.997 (0.752)	0.999 (0.599)	0.998 (0.595)
**Refinement**				
Resolution (Å)	37.79 – 2.71	43.48 - 1.9	44.84 - 1.7	39.22 - 1.501
No. reflections	34218	79457 (7865)	180004 (17923)	110607 (10829)
R_{work} / R_{free} (%)	17/22	17/20	18/20	18/21
No. atoms				
Protein	8684	6175	10033	4871
Solvent	27	639	1370	734
R.m.s. deviations:				
Bond lengths (Å)	0.01	0.004	0.006	0.005
Bond angles (°)	1.64	0.64	0.82	0.78
Ramachandran plot[#]				
Favored (%)	96.5	96.53	98.37	98.57

Allowed (%)	3.5	3.47	1.63	1.43
Outliers (%)	0	0.00	0.00	0.00
Rotamer outliers (%)	6.34	0.34	0.28	0.00
Clashscore	11.30	1.66	1.38	2.53
Average B-factor	45.95	38.86	34.19	29.63
Macromolecules	45.98	38.53	32.97	28.65
Solvent	37.28	42.03	43.13	36.17

R.m.s. deviation, root-mean square deviation.
Values in parentheses are for the highest resolution shell.
# Measured using Molprobity
